# *RNF213* p.Arg4810Lys (c.14429G>A) is associated with extracranial arterial stenosis

**DOI:** 10.1093/braincomms/fcaf049

**Published:** 2025-01-31

**Authors:** Daisuke Shimada, Satoru Miyawaki, Kaoru Nakanishi, Takashi Jono, Hibiku Maruoka, Takuya Kawai, Yoichi Harada, Takuji Kono, Koichiro Komatsubara, Jun Nakauchi, Yoshie Matsumoto, Kei Okada, Shogo Dofuku, Hiroki Hongo, Jun Mitsui, Yu Teranishi, Kenta Ohara, Daiichiro Ishigami, Yu Sakai, Hiroyuki Kawano, Akio Noguchi, Hirofumi Nakatomi, Nobuhito Saito, Teruyuki Hirano, Yoshiaki Shiokawa

**Affiliations:** Department of Neurosurgery, Kyorin University Faculty of Medicine, Tokyo 181-8611, Japan; Department of Neurosurgery, Faculty of Medicine, The University of Tokyo, Tokyo 113-8654, Japan; Department of Stroke and Cerebrovascular Medicine, Kyorin University Faculty of Medicine, Tokyo 181-8611, Japan; Department of Stroke and Cerebrovascular Medicine, Kyorin University Faculty of Medicine, Tokyo 181-8611, Japan; Department of Stroke and Cerebrovascular Medicine, Kyorin University Faculty of Medicine, Tokyo 181-8611, Japan; Department of Neurosurgery, Koyama Memorial Hospital, Ibaraki 314-0030, Japan; Department of Neurosurgery, Mito Brain Heart Center, Ibaraki 310-0004, Japan; Department of Neurosurgery, Mito Brain Heart Center, Ibaraki 310-0004, Japan; Department of Neurosurgery, Kugayama Hospital, Tokyo 157-0061, Japan; Department of Neurosurgery, Kanto Central Hospital, Tokyo 158-8531, Japan; Department of Neurosurgery, Kyorin University Faculty of Medicine, Tokyo 181-8611, Japan; Department of Neurosurgery, Kyorin University Faculty of Medicine, Tokyo 181-8611, Japan; Department of Neurosurgery, Faculty of Medicine, The University of Tokyo, Tokyo 113-8654, Japan; Department of Neurosurgery, Faculty of Medicine, The University of Tokyo, Tokyo 113-8654, Japan; Medical Genome Research Support Center, The University Tokyo Hospital, Tokyo 113-8654, Japan; Department of Neurosurgery, Faculty of Medicine, The University of Tokyo, Tokyo 113-8654, Japan; Department of Neurosurgery, Faculty of Medicine, The University of Tokyo, Tokyo 113-8654, Japan; Department of Neurosurgery, Faculty of Medicine, The University of Tokyo, Tokyo 113-8654, Japan; Department of Neurosurgery, Faculty of Medicine, The University of Tokyo, Tokyo 113-8654, Japan; Department of Stroke and Cerebrovascular Medicine, Kyorin University Faculty of Medicine, Tokyo 181-8611, Japan; Department of Neurosurgery, Kyorin University Faculty of Medicine, Tokyo 181-8611, Japan; Department of Neurosurgery, Kyorin University Faculty of Medicine, Tokyo 181-8611, Japan; Department of Neurosurgery, Faculty of Medicine, The University of Tokyo, Tokyo 113-8654, Japan; Department of Stroke and Cerebrovascular Medicine, Kyorin University Faculty of Medicine, Tokyo 181-8611, Japan; Department of Neurosurgery, Kyorin University Faculty of Medicine, Tokyo 181-8611, Japan

**Keywords:** *RNF213* p.Arg4810Lys (c.14429G>A), stroke, intracranial and extracranial artery stenosis, maximum intima-media thickness

## Abstract

*Ring finger protein 213* (*RNF213*) p.Arg4810Lys (c.14429G > A) is associated with intracranial artery stenosis; however, its association with extracranial artery stenosis remains unknown. We aimed to elucidate the clinical significance and association of *RNF213* p.Arg4810Lys with stroke subtypes, extracranial artery stenosis, and maximum intima-media thickness. A cohort of 600 patients with stroke prospectively collected over 1 year was assessed for the presence of *RNF213* p.Arg4810Lys. A total of 1202 patients served as controls. The association of *RNF213* p.Arg4810Lys with various stroke subtypes was studied. In sub-analyses, the association of *RNF213* p.Arg4810Lys with intracranial artery stenosis/extracranial artery stenosis and maximum intima-media thickness were assessed. *RNF213* p.Arg4810Lys was more common in patients with stroke (3.3%) than in those without stroke (1.1%). *RNF213* p.Arg4810Lys was significantly associated with stroke. Among various stroke subtypes, large-artery atherosclerosis, both due to intracranial artery stenosis and extracranial artery stenosis, was most significantly associated with *RNF213* p.Arg4810Lys. In the sub-analysis, intracranial artery stenosis-only, extracranial artery stenosis-only, and concurrent intracranial artery stenosis and extracranial artery stenosis groups were significantly associated with *RNF213* p.Arg4810Lys, regardless of stroke type (adjusted odds ratio, 3.72; 95% confidence interval, 1.30–10.60; *P=* 0.014, adjusted odds ratio, 7.04; 95% confidence interval, 1.51–32.77; *P=* 0.013, adjusted odds ratio, 11.68; 95% confidence interval, 4.25–32.07; *P* 0.001, respectively). *RNF213* p.Arg4810Lys was associated with increased maximum intima-media thickness, measured using carotid artery ultrasonography (multiple regression analysis β = 0.165; *P* = 0.004). These results were replicated in an independent validation cohort. In conclusion, *RNF213* p.Arg4810Lys increases the risk of stroke. In addition to intracranial artery stenosis, *RNF213* p.Arg4810Lys is associated with extracranial artery stenosis and maximum intima-media thickness. Evaluating *RNF213* p.Arg4810Lys may help predict the incidence and type of stroke.

## Teaser

Shimada *et al.* identified the genetic variant *RNF213* p.Arg4810Lys (c.14429G>A) as a significant risk factor for stroke in a Japanese population. The variant was found to be associated with large-artery atherosclerosis and its subtypes, including intracranial and extracranial arterial stenoses. Evaluating this genetic variant may help in predicting stroke incidence and type, particularly in East Asian populations.

## Graphical Abstract

**graphical abstract:**
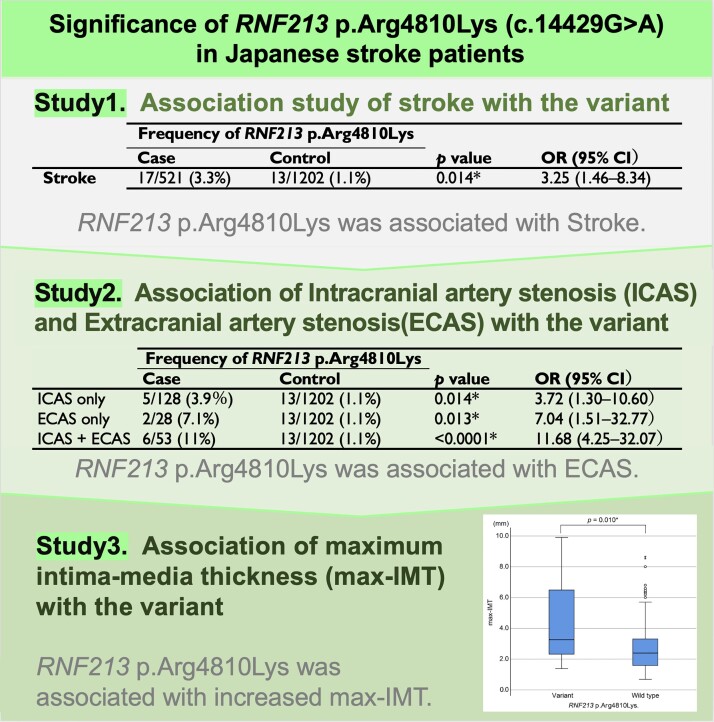

## Introduction

With advancements in genetic analysis research, genetic factors involved in the onset of various multifactorial diseases have been identified. Stroke is mainly caused by acquired factors, such as lifestyle-related diseases and genetic factors, which are associated with its pathogenesis.^1^ Recently, *ring finger protein 213* (*RNF213*) has been identified as a stroke-associated gene among East Asian populations.^2^

*RNF213* was originally identified as a susceptibility gene for moyamoya disease (MMD), mainly characterized by stenosis at the terminal portion of the intracranial internal carotid artery.^3,4^ MMD is highly prevalent in East Asian populations, including the Japanese.^5^ A genetic variant p.Arg4810Lys (c.14429G > A, rs112735431) of *RNF213*, present in approximately 80% of Japanese patients, is significantly associated with MMD in East Asian populations.^3,4^*RNF213* p.Arg4810Lys is also associated with common intracranial artery stenosis (ICAS) in these populations; it is found in approximately 20% of Japanese patients with ICAS.^6,7^ Furthermore, *RNF213* p.Arg4810Lys is a common risk factor for stroke in the Japanese population.^2^ The frequency of *RNF213* p.Arg4810Lys is approximately 1% in the general East Asian population, including the Japanese,^2,8^ but it is rarely found in other populations.^9^ Thus, *RNF213* p.Arg4810Lys is considered an important genetic factor for stroke; however, its frequency in the general stroke population remains unclear.

Controversy exists over which subtype of stroke is associated with the *RNF213* p.Arg4810Lys variant. Strokes are broadly categorized as either haemorrhagic or ischaemic. Ischaemic stroke is classified into cardioembolism (CE), large-artery atherosclerosis (LAA), and small vessel occlusion (SVO) subtypes based on the Trial of ORG 10172 in the Acute Stroke Treatment classification.^10^ Accordingly, genetic analysis of ischaemic stroke has been based on this classification.^1^ LAA is further classified into two types: one caused by atherosclerotic ICAS and the other one by atherosclerotic extracranial artery stenosis (ECAS).^10,11^ To date, few genetic analysis reports have been successful in distinguishing between these two LAA types. As previously mentioned, *RNF213* p.Arg4810Lys is significantly associated with ICAS, but analyses on the association between *RNF213* p.Arg4810Lys and ECAS are few.

Maximum intima-media thickness (max-IMT) is an index of arteriosclerosis in ECAS.^12^ A small number of studies have reported the genetic factors associated with max-IMT, such as endothelin receptor type A (*EDNRA*, rs17612742).^1^ However, the association between *RNF213* p.Arg4810Lys and max-IMT has not been reported.

Therefore, this study aimed to determine the frequency of the *RNF213* p.Arg4810Lys variant in the general stroke population, identify the stroke subtypes associated with *RNF213* p.Arg4810Lys, and investigate the association between *RNF213* p.Arg4810Lys and max-IMT. We separately analysed LAA caused by ICAS and LAA caused by ECAS to determine whether *RNF213* p.Arg4810Lys is associated with ECAS. For sub-analysis, an association study of ICAS or ECAS and *RNF213* p.Arg4810Lys was performed. Moreover, we investigated the association between *RNF213* p.Arg4810Lys and max-IMT.

## Materials and methods

### Patients (kyorin stroke cohort)

All consecutive stroke patients admitted to the Kyorin University Stroke Center from May 2020 to April 2021 were prospectively included. Exclusion criteria were patients who failed to provide consent to participate in the study, patients admitted with a suspected stroke but who had a different diagnosis based on examination during hospitalization, patients with epilepsy, and those with insufficient examination, as well as duplication due to patient readmission within a year. Blood samples and clinical data were obtained from patients who provided written informed consent.

### Diagnosis of stroke subtypes

Strokes are broadly categorized as either haemorrhagic or ischaemic. The TOAST classification indicates the following subtypes of ischaemic stroke: CE, LAA, SVO, other determined aetiologies (MMD, branch atheromatous disease [BAD], cerebral artery dissection, anomalous cerebral embolism, cerebral infarction due to aortic embolism, venous sinus thrombosis, and Trousseau syndrome), and undetermined aetiology.^10,11^ MMD was diagnosed based on the most recent diagnostic criteria.^13^ LAA was classified based on the responsible artery for cerebral infarction. Cerebral infarction due to ICAS and ECAS was categorized into LAA-ICAS and LAA-ECAS groups, respectively.

ICAS diagnosis was mainly based on the findings of magnetic resonance angiography and three-dimensional computed tomography angiography. The degree of ICAS was evaluated using the criteria of the Warfarin Aspirin Symptomatic Intracranial Disease trial.^14^ ICAS was classified into four types, namely, normal, < 50% stenosis, ≥ 50% stenosis, and complete occlusion. ECAS was evaluated using the European Carotid Surgery Trial method.^15^ It was defined as more than 50% stenosis in the extracranial internal carotid, common carotid, brachiocephalic and subclavian arteries, and proximal stenosis of the vertebral artery.

### Data collection

The following definitions of patient background factors were used. Hypertension was defined as systolic blood pressure ≥140 mmHg and diastolic blood pressure ≥90 mmHg or the use of antihypertensive medication.^3^ Dyslipidaemia was defined as the previous or current use of oral medications.^16^ Diabetes was defined as the use of hypoglycaemic drugs, self-discontinued use of medication, or diagnosis of diabetes during hospitalization.^17^ Hyperuricaemia was defined as the previous or current use of oral medication or the diagnosis of hyperuricaemia during hospitaliZation.^18^ Renal dysfunction was defined as the presence of documented kidney disease. Patients on dialysis were also included, because they had renal dysfunction.^19^ Atrial fibrillation was defined as the diagnosis of the disease after examination at any medical institution and catheter ablation.^20^ A history of tumours was defined as the diagnosis of malignant tumours (including treatment) by examination at a medical institution, regardless of tumour location.^21^ For family history, patients with a history of cardiovascular disease were considered.^22^ Peripheral arterial disease was defined as the diagnosis of arteriosclerosis obliterans (ASO), presence of leg ulcers or foot gangrene on examination, or previous treatment. Smoking history was defined as current or former smoking of ≥1 pack/year.^23^ A history of heavy drinking was defined as regular alcohol consumption of 300 g or more per week.^24^ A history of cerebral infarction was defined as cerebral infarction onset 1 month or more before admission. A history of cerebral haemorrhage was defined as the occurrence of cerebral haemorrhage more than 1 month before admission, not due to trauma. History of angina/myocardial infarction included patients with stable/unstable angina, myocardial infarction, or post-surgery.^22^ Hyper/hypothyroidism was defined as the diagnosis of a history of chronic conditions or conditions affecting thyroid function, the use of medications, or hospitaliZation due to the disease. Goitre was diagnosed based on imaging such as neck echocardiography.

### Evaluation of max-IMT

Max-IMT was evaluated using high-resolution B-mode carotid ultrasonography. Subjects were placed in a supine position on a bed, and the test was performed bilaterally along the longitudinal and cross-sectional axes. The max-IMT in the entire area was defined as the maximum measurable IMT in the scanned common carotid arteries, carotid bulb, internal carotid arteries, and external carotid artery areas for both sides.

### Genetic analysis of *RNF213* p.Arg4810Lys

We obtained genomic DNA from the peripheral blood leukocytes. Mutational analysis of exon 60, which includes the p.Arg4810Lys variant of *RNF213* (National Center for Biotechnology Information Reference Sequences NM_001256071 and NP_00124300), was performed using direct Sanger sequencing. The coding region that bears the p.Arg4810Lys variant of *RNF213* was amplified by PCR. We used the primers (forward: 5′-CTGCATCACAGGAAATGACACTG-3′, reverse: 5′-TGACGAGAAGAGGCTTTCAGACGA-3′, product size: 782 bp) for amplification and sequencing as reported previously.^25^ KAPA Taq Extra PCR Kit (Cat No. KK3009; Kapa Biosystems, Inc., Wilmington, MA, USA) was utilized. PCR-purified products were treated with illustra ExoProStar, Enzymatic PCR and Sequence Reaction Clean-up Kit (US78225; GE Healthcare Life Sciences, Chicago, IL, USA), and BigDye XTerminator™ Purification Kit (4376484; Applied Biosystems, Waltham, MA, USA) before sequencing. The Applied Biosystems 3730 DNA Analyzer (Thermo Fisher Scientific, Waltham, MA, USA) was used for Sanger sequencing.

As control data for the general population with the frequency of *RNF213* p.Arg4810Lys, we used genetic analysis data of healthy individuals (without a history of stroke) from the database of exome-sequenced samples at the Medical Genome Research Support Center, The University of Tokyo Hospital.

### Study 1: association of stroke with *RNF213* p.Arg4810Lys in the Kyorin stroke cohort

An association study with *RNF213* p.Arg4810Lys was performed in the Kyorin stroke cohort of consecutive patients. The association of *RNF213* p.Arg4810Lys with different stroke subtypes previously described was evaluated.

### Study 2: association of ICAS and ECAS with *RNF213* p.Arg4810Lys

The presence of ICAS and ECAS and their association with *RNF213* p.Arg4810Lys was analysed in the Kyorin stroke cohort of consecutive patients. Patients with arterial stenosis that was difficult to evaluate (those with CE and MMD) were excluded. The analysis was validated in an independent cohort. The validation cohort consisted of patients who visited four other hospitals (Koyama Memorial Hospital, Mito Brain Heart Center/Brain-pia, Kugayama Hospital, and Kanto Central Hospital) during the study period. The patients were evaluated for the prevalence of ICAS and ECAS using the same criteria. To evaluate ICAS and ECAS separately, we divided the patients into ICAS-only, ECAS-only, and concurrent ICAS and ECAS groups. The control group of patients without stenosis from the same hospitals was also included.

### Study 3: association of max-IMT with *RNF213* p.Arg4810Lys

The association between max-IMT and *RNF213* p.Arg4810Lys was investigated in the Kyorin stroke cohort of consecutive patients. Patients with arterial stenosis that was difficult to evaluate (those with CE and MMD) were excluded. Risk factors for max-IMT were also evaluated. The analysis was validated in an independent cohort.

### Ethics approval

This study was approved by the Human Genome Genetic Analysis Research Ethics Committee of Kyorin University School of Medicine (approval number: 771), Faculty of Medicine, The University of Tokyo (approval number: G10026), and the ethics committees of Koyama Memorial Hospital (approval number: 0200316-1), Mito Brain Heart Center/Brain-pia (approval number: 2020-0004), Kugayama Hospital (approval number: 2020-E004), and Kanto Central Hospital (approval number: R2-25). We performed this study in accordance with the Declaration of Helsinki.

### Statistical analysis

All statistical analyses were performed using IBM SPSS version 26 (IBM, Armonk, NY, USA). The effect of the background factors according to the presence or absence of *RNF213* p.Arg4810Lys in the two groups was measured using the two-tailed Fisher’s exact test and Mann–Whitney *U* test (for age). The association between *RNF213* p.Arg4810Lys and each stroke type was analysed using logistic regression analysis, after adjusting for age and sex. In our logistic regression analysis, sex was included as a binary variable, and age was treated as a continuous variable to adjust for these demographic factors. Logistic regression analysis was also used to determine the association of *RNF213* p.Arg4810Lys with ICAS and ECAS, with adjustments for age and sex. The association between *RNF213* p.Arg4810Lys and max-IMT was analysed using the Mann–Whitney *U* test. A single regression analysis was initially performed to verify risk factors for max-IMT. Those with significant differences were analysed using multiple regression analysis. Statistical significance was set at *P<* 0.05.

## Results

### Study 1: association of stroke with *RNF213* p.Arg4810Lys in the Kyorin stroke cohort

**Figure 1:**
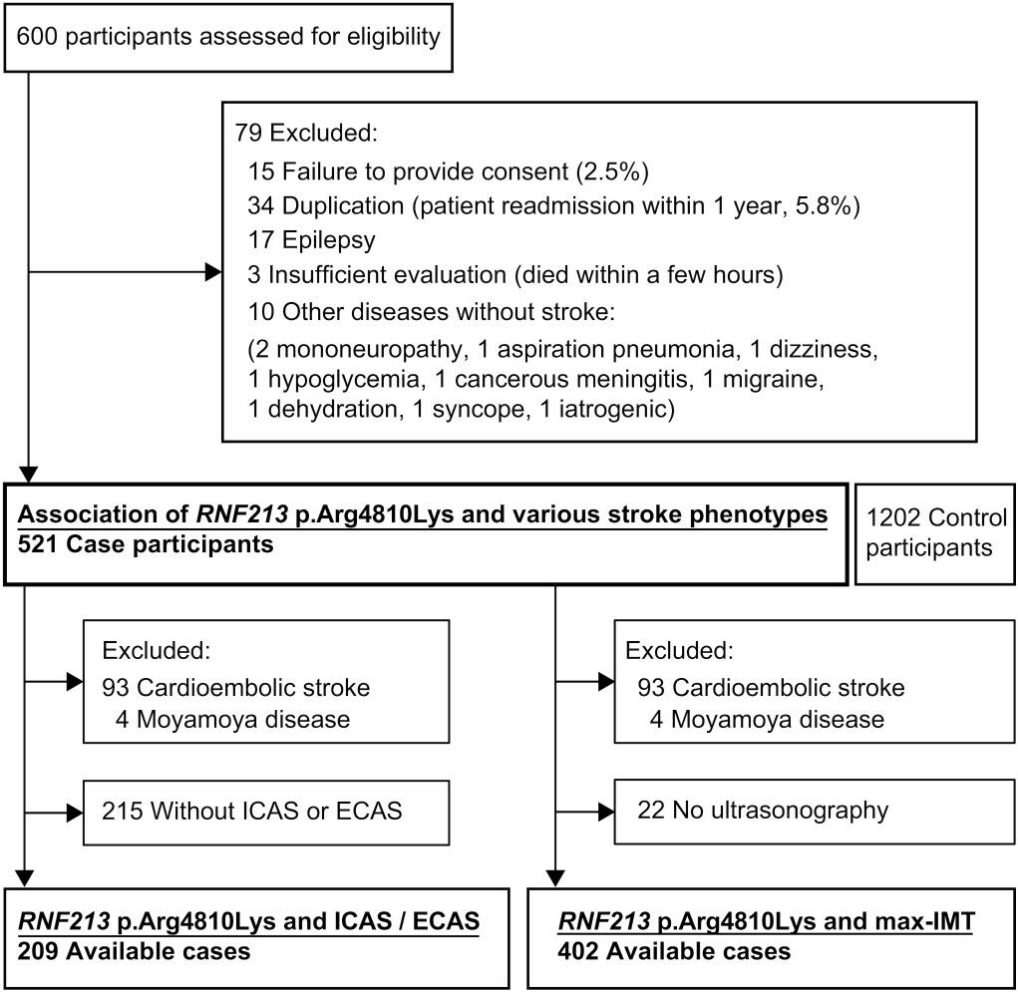
**Flowchart of the Kyorin cohort showing the association of the *RNF213* p.Arg4810Lys variant with ICAS/ECAS and max-IMT.** ECAS, extracranial artery stenosis; ICAS, intracranial artery stenosis; max-IMT, maximum intima-media thickness.

Of the 600 hospitalized patients at our Stroke Center, 521 were included in the Kyorin stroke cohort. Data of 1202 patients with no history of stroke or heart disease from the database of the Medical Genome Research Support Center were used as the control (Fig. 1).

In the patient group, the average age was 73 (40–72) years, and 42% (218/521) were women. *RNF213* p.Arg4810Lys heterozygous variant (GA) and *RNF213* p.Arg4810Lys wild-type (GG type) were detected in 17 and 504 patients, respectively. The control group was aged 49 (35–62) years, and 52% (628/1202) were women. *RNF213* p.Arg4810Lys heterozygous variant (GA) was detected in 13 patients, and *RNF213* p.Arg4810Lys wild-type (GG) in 1189 patients. The *RNF213* p.Arg4810Lys homozygous variant (AA) was absent in both groups (Table 1).

**Table 1:** Clinical characteristics of the patients

	Total	Stroke subtype						
		CE	LAA	SVO	Others	ICH	SAH	TIA
*N*	521	93 (18%)	97 (19%)	59 (11%)	128 (24%)	129 (25%)	5 (0.9%)	10 (1.9%)
Age (years)	73 (40–72)	80 (75–86)	76 (69–84)	74 (65–85)	71 (63–82)	69 (58–81)	55 (56–63)	68 (68–82)
Sex (female)	217 (42%)	42 (46%)	28 (29%)	25 (42%)	60 (47%)	55 (42%)	3 (60%)	6 (60%)
*RNF213*.Arg4810Lys (GG/GA/AA)	504/17/0	91/1/0	87/10/0	59/0/0	124/3/0	128/3/0	5/0/0	69/0/0
Hypertension	381 (73%)	70 (76%)	79 (81%)	45 (76%)	87 (69%)	93 (71%)	1 (20%)	6 (60%)
Diabetes mellitus	116 (22%)	17 (18%)	33 (34%)	18 (31%)	27 (21%)	20 (15%)	0 (0%)	1 (10%)
Dyslipidaemia	177 (34%)	29 (32%)	50 (52%)	18 (31%)	39 (31%)	36 (27%)	1 (20%)	4 (40%)
Hyperuricaemia	50 (9.6%)	9 (9.7%)	8 (8.2%)	2 (3.4%)	20 (16%)	10 (7.8%)	1 (20%)	0 (0%)
Renal dysfunction	43 (8.2%)	10 (11%)	11 (11%)	4 (6.8%)	5 (3.9%)	12 (9.3%)	0 (0%)	1 (10%)
PAD	9 (1.7%)	2 (2.2%)	3 (3.1%)	1 (1.7%)	1 (0.8%)	2 (1.6%)	0 (0%)	0 (0%)
Thyroid disease	37 (7.1%)	10 (11%)	3 (3.1%)	5 (8.5%)	8 (6.3%)	10 (7.8%)	0 (0%)	1 (10%)
Previous CAD	53 (10%)	10 (11%)	17 (18%)	6 (10%)	10 (7.8%)	9 (7.0%)	0 (0%)	1 (10%)
Previous CI	105 (20%)	28 (30%)	18 (19%)	13 (22%)	23 (18%)	22 (17%)	0 (0%)	1 (10%)
Previous ICH	39 (7.5%)	6 (6.5%)	8 (8.2%)	3 (5.1%)	3 (2.3%)	19 (15%)	0 (0%)	0 (0%)
Previous tumour	113 (22%)	24 (26%)	21 (22%)	12 (20%)	27 (21%)	26 (20%)	0 (0%)	3 (30%)
Family history	181 (35%)	31 (34%)	35 (36%)	18 (31%)	52 (41%)	40 (31%)	0 (0%)	5 (50%)
Smoking (current or former)	245 (47%)	45 (49%)	55 (57%)	30 (51%)	55 (43%)	54 (41%)	1 (20%)	5 (50%)
Heavy drinkers	64 (12%)	8 (9%)	14 (14%)	7 (12%)	17 (13%)	17 (13%)	1 (20%)	0 (0%)
BMI (kg/m^2^)	22.8	22.7	22.6	22.9	24.1	22.3	22.0	24.1

Values are expressed as *n* (%), median age [Quartile 1 and Quartile 3].

CE, cardioembolism; LAA, large-artery atherosclerosis; SVO, small vessel occlusion; ICH, intracerebral haemorrhage; SAH, subarachnoid haemorrhage; TIA, transit ischaemic attack; PAD, peripheral artery disease; CAD, coronary artery disease; CI, cerebral infarction; BMI, body mass index.

The frequency of the *RNF213* p.Arg4810Lys variant was 3.3% (17/521) in the patient group and 1.1% (13/1202) in the control group. Logistic regression analysis adjusted for age and sex showed that *RNF213* p.Arg4810Lys was significantly associated with stroke (adjusted odds ratio [aOR], 3.25; 95% confidence interval [CI], 1.46–8.34; *P=* 0.014) (Table 2).

**Table 2:** Association of stroke subtype with *RNF213* p.Arg4810Lys

	Frequency of *RNF213* p.Arg4810Lys		*P* value	OR (95% CI）
	Case	Control		
Total	17/521 (3.3%)	13/1202 (1.1%)	0.014*	3.25 (1.46–8.34)
Stroke subtype				
CE	1/93 (1.1%)		0.961	0.94 (0.96–9.27)
LAA	10/97 (10.3%)		<0.001*	14.55 (4.41–48.05)
SVO	0/59 (0%)		0.999	0.00
Others (non-MMD)	1/124 (0.8%)		0.814	0.77 (0.09–6.73)
Others (MMD)	2/4 (50.0%)		<0.001*	77.55 (9.56–628.76)
ICH	3/131 (2.3%)		0.309	2.10 (0.50–8.72)
SAH	0/5 (0%)		0.999	0.00
TIA	0/10 (0%)		0.999	0.00
LAA subtype				
LAA-ICAS	7/69 (10.1%)		<0.001*	18.52 (5.58–61.50)
LAA-ECAS	3/28 (10.7%)		<0.001*	10.98 (2.94–40.94)

Statistically significant differences were determined using multiple logistic regression adjusted for age and sex.

OR, odds ratio; CI, confidence interval; CE, cardioembolism; LAA, large-artery atherosclerosis; SVO, small vessel occlusion; MMD, moyamoya disease; ICH, intracerebral haemorrhage; SAH, subarachnoid haemorrhage; TIA, transient ischaemic attack; ICAS, intracranial artery stenosis; ECAS, extracranial artery stenosis.

^*^Statistical significance: *P* < 0.05.

Stroke in patients with *RNF213* p.Arg4810Lys was characterized by a history of dyslipidaemia (59% versus 36%, *P=* 0.036) and coronary artery disease (29% versus 13%, *P=* 0.022). No differences in other factors, including age and sex, were found in patients with or without *RNF213* p.Arg4810Lys (Supplemental Table 1).

LAA, CE, and intracerebral haemorrhage (ICH) were present in 19% (97/521), 18% (93/521), and 25% (131/521), respectively, of the patients in the patient group. *RNF213* p.Arg4810Lys was detected in 1.1% of the patients (1/93) with CE, 10.3% of the patients (10/97) with LAA, 0% of the patients (0/59) with SVO, 50% of the patients (2/4) with MMD, 0.8% of the patients (1/124) with other types excluding MMD, and 2.3% of the patients (3/131) with ICH.

Among other determined types of disease, *RNF213* p.Arg4810Lys was found in patients with cerebral artery dissection (6.7%; 1/15) but not in those with perforating branch infarction, including SVO and BAD (0/84) and subarachnoid haemorrhage (0/5). Among different stroke subtypes, MMD and LAA were significantly associated with *RNF213* p.Arg4810Lys (aOR, 77.55; 95%CI, 9.56–628.76; *P<* 0.001, aOR, 14.55; 95% CI, 4.41–48.05; *P* < 0.001, respectively). CE, SVO, others, and ICH showed no significant association (Table 2 and Supplemental Table 2).

We analysed the differences in background factors depending on the presence or absence of *RNF213* p.Arg4810Lys in LAA (*RNF213* p.Arg4810Lys variant, 10 patients; *RNF213* p.Arg4810Lys wild-type, 87 patients) and found no significantly associated factors in both groups (Supplemental Table 3).

LAA was categorized based on the site of the responsible vessel; the LAA-ICAS group comprised 69 patients, and the LAA-ECAS group 28 patients. The percentage of patients with *RNF213* p.Arg4810Lys was almost equal in the LAA-ICAS (10.1% [7/69]) and LAA-ECAS (10.7% [3/28]) groups. After adjustment for age and sex, both groups were significantly associated with *RNF213* p.Arg4810Lys compared with the control group (aOR, 18.52; 95% CI, 5.58–61.50; *P<* 0.001, aOR, 10.98; 95% CI, 2.94–40.94; *P<* 0.001, respectively). *RNF213* p.Arg4810Lys was significantly associated with both ICAS and ECAS in LAA.

### Study 2: association of ICAS and ECAS with *RNF213* p.Arg4810Lys

Regardless of stroke subtypes, 209 patients with ICAS and/or ECAS were examined in the Kyorin stroke cohort. Of these patients, 13 had the *RNF213* p.Arg4810Lys variant. Stenosis in the anterior circulation was present in 100% (13/13) of the patients with the *RNF213* p.Arg4810Lys variant and 74% (146/196) of those with the wild-type. The middle cerebral artery was the most common site of stenosis. Three or more tandem lesions were more common in patients with *RNF213* p.Arg4810Lys (38% versus 6.8%; Supplemental Table 4).

To further evaluate ICAS and ECAS separately, we divided the patients into those with ICAS-only, ECAS-only, and concurrent ICAS and ECAS groups. The frequency of *RNF213* p.Arg4810Lys was 3.9% (5/128) in the ICAS-only group, 7.1% (2/28) in the ECAS-only group, and 11% (6/53) in the concurrent ICAS and ECAS group. After adjusting for age and sex, a logistic regression analysis was performed. *RNF213* p.Arg4810Lys was significantly associated with ICAS-only, ECAS-only, and concurrent ICAS and ECAS (aOR, 3.72; 95% CI, 1.30–10.60; *P=* 0.014, aOR, 7.04; 95% CI, 1.51–32.77; *P=* 0.013, aOR, 11.68; 95% CI, 4.25-32.07; *P* < 0.0001, respectively; Table 3).

**Table 3:** Association of ICAS and ECAS with *RNF213* p.Arg4810Lys

	Frequency of *RNF213* p.Arg4810Lys		*P* value	OR (95% CI)
	Case	Control		
ICAS-only	5/128 (3.9%)	13/1202 (1.1%)	0.014*	3.72 (1.30–10.60)
ECAS-only	2/28 (7.1%)	13/1202 (1.1%)	0.013*	7.04 (1.51–32.77)
ICAS + ECAS	6/53 (11%)	13/1202 (1.1%)	<0.0001*	11.68 (4.25–32.07）

Statistically significant differences were determined using multiple logistic regression adjusted for age and sex.

ICAS, intracranial artery stenosis; ECAS, extracranial artery stenosis; CI, confidence interval; OR, odds ratio.

^*^Statistical significance: *P* < 0.05.

Of the 391 patients identified in the validation cohort from four other hospitals, 229 with ICAS and ECAS were eligible for the study (Supplemental Fig. 1). These include 159 patients with ICAS, 24 with ECAS, and 46 with concurrent ICAS and ECAS (Supplemental Table 4). A total of 131 control samples were also collected. The frequency of *RNF213* p.Arg4810Lys was 6.2% (10/159) in the ICAS-only group, 8.3% (2/24) in the ECAS-only group, and 22% (10/46) in the concurrent ICAS and ECAS group, compared with 0.8% (1/131) in the control group. *RNF213* p.Arg4810Lys was significantly associated with ICAS-only, ECAS-only, and concurrent ICAS and ECAS (aOR, 10.08; 95%CI, 1.28–79.13; *P* = 0.007, aOR, 10.56; 95%CI, 1.33–83.59; *P* = 0.026, and aOR, 32.75; 95%CI, 4.02–267.12; *P* < 0.001, respectively) (Supplemental Table 5).

*RNF213* p.Arg4810Lys was significantly associated with concurrent ICAS and ECAS in the Japanese stroke group. The percentage of patients with *RNF213* p.Arg4810Lys was highest in the concurrent ICAS and ECAS groups.

### Study 3: association of max-IMT with *RNF213* p.Arg4810Lys

**Figure 2:**
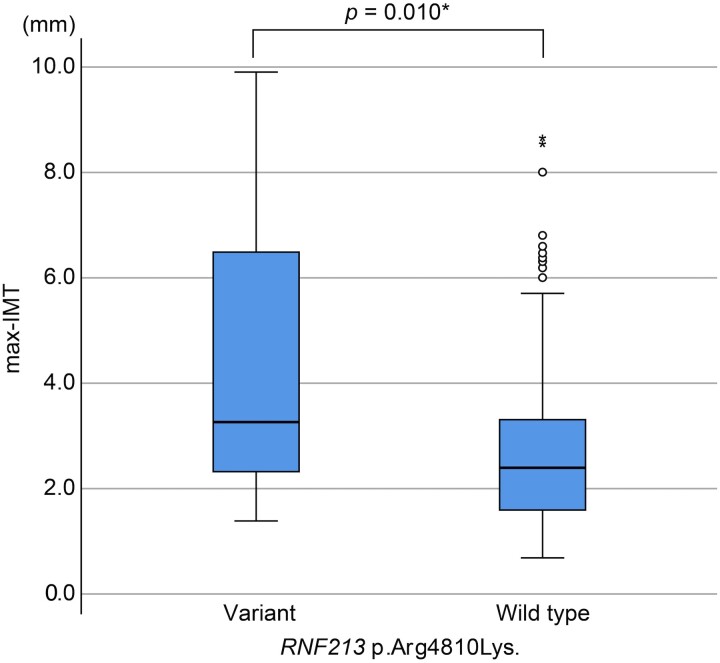
**Max-IMT of carotid artery by *RNF213* p.Arg4810Lys genotypes in the Kyorin cohort.** Box plot shows the significantly increased max-IMT of the carotid artery in the *RNF213* p.Arg4810Lys variant group (*N* = 14) compared to the wild-type group (*N* = 388). Statistical analysis was performed using the Mann–Whitney *U* test, with *P < 0.05 indicating significance. Max-IMT, maximum intima-media thickness.

A total of 402 patients who underwent carotid ultrasonography were eligible for evaluation in the Kyorin stroke cohort. The max-IMT was 4.2 ± 2.5 mm (range, 2.0–9.9) in the *RNF213* p.Arg4810Lys variant group and 2.7 ± 1.4 mm (range, 0.8–8.6) in the wild-type group. Max-IMT was significantly thicker in the *RNF213* p.Arg4810Lys variant group (*P* = 0.010; Fig. 2). Association analysis with risk factors showed that max-IMT was affected by age (β = 0.324, *P* < 0.0001), sex (β = 0.173, *P* < 0.0001), *RNF213* p.Arg4810Lys (β = 0.195, *P* < 0.0001), history of hypertension (β = 0.139, *P* = 0.005), history of diabetes (β = 0.176, *P* < 0.0001), history of hyperlipidaemia (β = 0.187, *P* < 0.0001), history of renal dysfunction (β = 0.142, *P* = 0.007), peripheral artery disease (β = 0.105, *P* = 0.034), history of coronary artery disease (β = 0.230, *P* < 0.0001), and smoking (β = 0.180, *P* < 0.0001). In multiple regression analysis, max-IMT was associated with age (β = 0.348, *P* < 0.0001), sex (β = 0.179, *P* < 0.0001), *RNF213* p.Arg4810Lys (β = 0.179, *P* < 0.0001), history of coronary artery disease (β = 0.124, *P* = 0.007), and smoking (β = 0.127, *P* = 0.011) (Table 4).

**Table 4:** Single and multiple regression analyses of risk factors for max-IMT

Risk factor	Single regression analysis			Multiple regression analysis		
	β	SE	*P* value	β	SE	*P* value
Age	0.324	0.005	<0.0001*	0.348	0.005	<0.0001*
Sex (female)	0.173	0.145	<0.0001*	0.179	0.148	<0.0001*
*RNF213* p.Arg4810Lys	0.195	0.389	<0.0001*	0.165	0.347	<0.0001*
Hypertension	0.139	0.160	0.005*	0.042	0.144	0.339
Diabetes mellitus	0.176	0.166	<0.0001*	0.061	0.155	0.180
Dyslipidaemia	0.187	0.149	<0.0001*	0.083	0.139	0.070
Hyperuricaemia	0.056	0.235	0.263			
Renal dysfunction	0.142	0.249	0.007*	0.044	0.224	0.321
PAD	0.105	0.596	0.034*	0.064	0.525	0.142
Thyroid disease	−0.084	0.300	0.092			
Previous CAD	0.230	0.226	<0.0001*	0.124	0.1160	0.007*
Previous CI	−0.011	0.184	0.823			
Previous ICH	−0.043	0.261	0.390			
Previous tumour	0.018	0.176	0.714			
Family history	−0.076	0.151	0.128			
Smoking (current or former)	0.180	0.143	<0.0001*	0.127	0.145	0.011*
Heavy drinkers	0.081	0.209	0.102			
BMI	−0.033	0.015	0.530			

Statistically significant differences were determined using multiple regression analysis.

Max-IMT, maximum intima-media thickness; PAD, peripheral artery disease; CAD, coronary artery disease; CI, cerebral infarction; ICH, intracranial haemorrhage; BMI, body mass index.

^*^Statistical significance: *P* < 0.05.

Of the 391 patients in the validation cohort, 274 who underwent carotid ultrasonography were eligible for the study (Supplemental Fig. 1). The max-IMT in the *RNF213* p.Arg4810Lys group was 3.2 ± 1.8 mm (range, 1.4–7.8), and that in the wild-type group was 2.4 ± 1.3 mm (range, 0.4–7.1). The max-IMT was significantly thicker in the *RNF213* p.Arg4810Lys group than in the wild-type group (*P* = 0.009; Supplemental Fig. 2). Single and multiple regression analyses revealed that the max-IMT in the validation group was significantly associated with age, sex, *RNF213* p.Arg4810Lys, and a history of coronary artery disease (Supplemental Table 6).

Overall, max-IMT was significantly thicker in the *RNF213* p.Arg4810Lys group than in the wild-type group, and *RNF213* p.Arg4810Lys was significantly associated with max-IMT; this association was also found in the independent validation cohort.

## Discussion

This study explored the clinical significance and association of *RNF213* p.Arg4810Lys with stroke subtypes, ECAS, and max-IMT. We found that *RNF213* p.Arg4810Lys was present in 3.3% of the general Japanese stroke cohort and was a significant risk factor for stroke. Among the various subtypes of stroke, LAA was significantly associated with *RNF213* p.Arg4810Lys. Both LAA-ICAS and LAA-ECAS were significantly associated with *RNF213* p.Arg4810Lys. Furthermore, ICAS, ECAS, and max-IMT were significantly associated with *RNF213* p.Arg4810Lys.

*RNF213* p.Arg4810Lys has been reported to be associated with stroke in East Asian populations.^2^ However, the frequency of *RNF213* p.Arg4810Lys in the Japanese population with stroke is unclear. In the present study, *RNF213* p.Arg4810Lys was a significant risk factor for stroke in 3.3% of strokes in a 1-year prospective cohort from a single Japanese stroke centre. Although the prevalence of the *RNF213* p.Arg4810Lys variant is relatively low, it may still represent a significant genetic risk factor for East Asians, given that its odds ratio is higher than that of other stroke-related variants reported in large-scale genome-wide association studies (GWAS).^1^

*RNF213* p.Arg4810Lys has not been identified as a stroke-related gene in previous GWAS of stroke.^1^*RNF213* p.Arg4810Lys is found in approximately 1% of the general population in East Asian populations, including the Japanese, but it is rarely found in other populations, including European populations.^9^ Previous GWAS of stroke focused on genetic variants with a minor allele frequency of >1% in European populations and did not include *RNF213* p.Arg4810Lys variant in their analyses.^1^ Therefore, *RNF213* p.Arg4810Lys has not been included in previous stroke GWAS and has not been identified as a significant stroke-related genetic variant, and its significance has not been verified. Genetic analysis studies focusing on East Asian-specific gene variants, such as *RNF213* p.Arg4810Lys, may identify stroke-associated gene variants unique to populations.

*RNF213* p.Arg4810Lys has been reported to be associated with LAA, but its detailed association with the stroke subtype has not been elucidated. This paper presents a novel study that distinguishes between LAA-ICAS and LAA-ECAS based on the responsible vessel. We found that *RNF213* p.Arg4810Lys was associated with both LAA-ICAS and LAA-ECAS.

We further examined the proportion of our cohort with ICAS and ECAS, independent of stroke subtype, and analysed the association with *RNF213* p.Arg4810Lys. Although the association between *RNF213* p.Arg4810Lys and ICAS was reported in numerous studies.^6,7,26-29^ Similar to previous reports, we found an association between ICAS and *RNF213* p.Arg4810Lys.^6,7^ Several reports have demonstrated an association between ECAS and the *RNF213* p.Arg4810Lys variant. A study on Chinese patients with stroke or TIA showed that the *RNF213* p.Arg4810Lys variant is associated with LAA, anterior circulation ICAS, and ECAS.^30^ Another study on Japanese participants reported that the *RNF213* p.Arg4810Lys variant was associated with a smaller external diameter of the carotid artery compared to the GG genotype,^31^ suggesting some impact on the carotid artery. The novelty of our study lies in two key points. First, we examined the association between ECAS and *RNF213* p.Arg4810Lys in consecutive cases treated at our stroke centre over a one-year period. Second, we measured max-IMT using carotid ultrasound, and quantitatively clarified the association between cervical carotid artery stenosis and the *RNF213* p.Arg4810Lys variant.

Furthermore, *RNF213* p.Arg4810Lys was significantly associated with ECAS and with max-IMT. The pathogenic mechanisms of *RNF213* remain unclear, and its direct relationship with IMT and vascular pathology is yet to be fully elucidated.

*RNF213* is located on the long arm of chromosome 17 and encodes a large protein with 5207 amino acids. RNF213 is a protein with two AAA + ATPase domains and one RING finger ubiquitin ligase domain.^3,4^ AAA + ATPase is involved in the formation of hexamers, conformational changes associated with ATP hydrolysis, and their contributions to various intracellular transports.^32^*RNF213* p.Arg4810Lys is located in the C-terminal region of the RING domain.^3,4^ Various analyses have been conducted at the cellular and animal levels to determine the role of *RNF213* in the vascular system.

At the cellular level, studies using induced pluripotent stem cells from patients with MMD suggest that *RNF213* p.Arg4810Lys is involved in the dysfunction of vascular endothelial cells.^33^ In an animal model, knockdown of *RNF213* in zebrafish causes abnormal development of blood vessels in the head and neck regions, suggesting a function related to vascular development.^3^ Mouse studies have reported that knockout and knockin of *RNF213* p.Arg4810Lys do not result in intracranial vascular abnormalities.^34,35^*RNF213* p.Arg4810Lys alone does not cause intracranial vascular abnormalities,^36^ suggesting that additional factors are required for disease development. Additionally, RNF213 acts on lipid droplet stabiliZation in cells other than the vascular system, suggesting its potential role in regulating lipid metabolism.^37^

Recently, *RNF213* has been shown to be associated with immune function, which attracted attention.^38^*RNF213* is widely expressed not only in the blood vessels but also throughout the body and is particularly abundant in immune system organs and cells such as the spleen and lymphocytes.^4^*RNF213* expression is regulated by INF-β, an inflammatory signal, and the NFκB signalling pathway acts downstream of *RNF213.* The relationship between inflammation and immunity has also been reported.^36,39^ The association between inflammation and *RNF213* p.Arg4810Lys may be related to the association of *RNF213* p.Arg4810Lys with ICAS, ECAS, and increased max-IMT.

*RNF213* p.Arg4810Lys is known to be associated not only with ICAS and MMD but also with systemic vascular diseases, such as coronary artery stenosis, pulmonary artery stenosis, pulmonary hypertension, and renal artery stenosis.^40-47^ In a genome-wide association study including approximately 170 000 Japanese individuals, the *RNF213* variant was newly identified as being associated with coronary artery disease.^40^ Additionally, we previously reported an association between *RNF213* p.Arg4810Lys and angina pectoris through a phenome-wide association study.^45^ This study confirms similar results, suggesting that *RNF213* p.Arg4810Lys is also associated with systemic vascular conditions.

The limitations of this study are as follows. Although this study included a prospectively collected cohort, the number of patients in both the LAA-ICAS and LAA-ECAS groups was limited. This finding is particularly relevant to East Asian populations, including the Japanese population. We acknowledge that our sample size was relatively small, and larger, multicentre, and multi-year studies are necessary to validate and expand upon our findings. An analysis with a larger sample size is warranted to elucidate the association between *RNF213* p.Arg4810Lys and LAA-ECAS. The mechanism by which *RNF213* p.Arg4810Lys is associated with increased IMT has not yet been analysed. Further functional analysis of RNF213 is needed.

In conclusion, this prospective observational study is the first to report that *RNF213* p.Arg4810Lys is significantly associated with stroke in 3.3% of the general stroke cases. A sub-analysis of LAA revealed that *RNF213* p.Arg4810Lys was significantly associated with both LAA-ICAS and LAA-ECAS. Furthermore, *RNF213* p.Arg4810Lys was significantly associated with ICAS, ECAS, and max-IMT. These findings suggest that *RNF213* p.Arg4810Lys might be considered as a potential predictor of stroke incidence and type in the Japanese population, though further studies are necessary to confirm its reliability.

## Supplementary Material

fcaf049_Supplementary_Data

## Data Availability

Data are available on reasonable request. The authors confirm that the data supporting the findings of this study will be shared by request from any qualified investigator.
