# Supplementary material for: RNF213 p.Arg4810Lys (c.14429G>A) is associated with extracranial arterial stenosis
Source: Brain Commun. 2025 Jan 31;7(1):fcaf049. doi: 10.1093/braincomms/fcaf049 (PMC11829204; doi:10.1093/braincomms/fcaf049)
Supplement: fcaf049_Supplementary_Data [file fcaf049_supplementary_data.docx]

***Supplementary material***

***RNF213* p.Arg4810Lys (c.14429G>A) is associated with extracranial arterial stenosis**

Daisuke Shimada, MD, PhD^1†^ Satoru Miyawaki, MD, PhD^2†^ Kaoru Nakanishi, MD^3^ Takashi Jono, MD^3^ Hibiku Maruoka, MD^3^ Takuya Kawai, MD^5^ Yoichi Harada, MD, PhD^6^ Takuji Kono, MD, PhD^6^ Koichiro Komatsubara, MD, PhD^7^ Jun Nakauchi, MD, PhD^8^ Yoshie Matsumoto, MD^1^ Kei Okada, MD^1^ Shogo Dofuku, MD, PhD^2^ Hiroki Hongo, MD, PhD^2^ Mitsui Jun, MD, PhD^4^ Yu Teranishi, MD, PhD^2^ Kenta Ohara, MD, PhD^2^ Daiichiro Ishigami, MD, PhD^2^ Yu Sakai, MD^2^ Hiroyuki Kawano, MD, PhD^3^ Akio Noguchi, MD, PhD^1^ Hirofumi Nakatomi, MD, PhD^1^ Nobuhito Saito, MD, PhD^2^ Teruyuki Hirano, MD, PhD^3^ and Yoshiaki Shiokawa, MD, PhD^1^

**Correspondence to:** Satoru Miyawaki

Department of Neurosurgery, Faculty of Medicine, The University of Tokyo, 7-3-1 Hongo, Bunkyo-ku, Tokyo 113-8655, Japan

E-mail: [smiya-nsu@m.u-tokyo.ac.jp](mailto:smiya-nsu@m.u-tokyo.ac.jp)

**Supplementary Table 1 Baseline characteristics of stroke patients with or without *RNF213* p.Arg4810Lys**

|  | ***RNF213* p.Arg4810Lys**  **(N=17)** | **Wild type**  **(N=504)** | ***p* value** |  | ***RNF213* p.Arg4810Lys**  **(N=17)** | **Wild type**  **(N=504)** | ***p* value** |
| --- | --- | --- | --- | --- | --- | --- | --- |
| Age (years) | 71.5 (63–79) | 72.8 (65–84) | 0.519 | Previous tumour | 4 (24%) | 138 (30%) | 1.000 |
| Sex (female) | 7 (41%) | 227 (45%) | 0.809 | Living alone | 1 (5.9%) | 94 (19%) | 0.334 |
| Hypertension | 13 (77%) | 369 (74%) | 1.000 | Family history | 10 (59%) | 192 (41%) | 0.127 |
| Diabetes mellitus | 5 (29%) | 112 (25%) | 0.545 | Smoking  (current or former) | 7 (47%) | 236 (51%) | 0.625 |
| Dyslipidaemia | 10 (59%) | 166 (36%) | 0.036* | Heavy drinkers | 1 (5.9%) | 73 (14%) | 0.489 |
| Hyperuricemia | 3 (18%) | 46 (13%) | 0.210 | Previous CAD | 5 (29%) | 48 (13%) | 0.022* |
| Atrial fibrillation | 2 (12%) | 102 (24%) | 0.546 | Previous CI | 3 (18%) | 101 (23%) | 1.000 |
| Renal dysfunction | 1 (5.9%) | 45 (13%) | 1.000 | Previous ICH | 2 (12%) | 38 (11%) | 0.382 |
| Thyroid disease | 2 (12%) | 34 (6.7%) | 0.331 | Antiplatelet | 6 (36%) | 112 (23%) | 0.421 |
| PAD | 1 (5.9%) | 8 (5.5%) | 1.000 | Anticoagulant | 2 (12%) | 82 (17%) | 1.000 |

Statistically significant differences in background factors between the two groups were assessed using Fisher’s exact test and the Mann–Whitney U test (for age). *Statistical significance: *p*<0.05. Median age [Quartile 1 and Quartile 3].

Max‑IMT, maximum intima-media thickness; PAD, peripheral artery disease; CAD, coronary artery disease; CI, cerebral infarction; ICH, intracranial haemorrhage

**Supplementary Table 2 Other aetiologic subgroups**

| **Other subtypes** | **N** | **Frequency of *RNF213* p.Arg4810Lys** |
| --- | --- | --- |
| **Determined aetiology (N=87)** | | 3(3.4%) |
| Moyamoya disease | 4 (3.1%) | 2 (50%) |
| Branch atheromatous disease | 25 (20%) | 0 (0%) |
| Cerebral artery dissection | 15 (12%) | 1 (6.7%) |
| Trousseau syndrome | 13 (10%) | 0 (0%) |
| Aortic cerebral embolism | 8 (6.3%) | 0 (0%) |
| Paradoxical embolism | 7 (5.5%) | 0 (0%) |
| Cerebral vein thrombosis | 4 (3.1%) | 0 (0%) |
| After radiation therapy | 2 (1.6%) | 0 (0%) |
| Antiphospholipid syndrome | 2 (1.6%) | 0 (0%) |
| Pulmonary arteriovenous fistula | 1 (0.8%) | 0 (0%) |
| Neurofibromatosis type I | 1 (0.8%) | 0 (0%) |
| Fibromuscular dysplasia | 1 (0.8%) | 0 (0%) |
| Other causes | 4 (3.1%) | 0 (0%) |
| **Undetermined aetiology (N=41)** | | 0 (0%) |

**Supplementary Table 3 Differences in the background factors of patients with or without *RNF213* p.Arg4810Lys in large artery atherosclerosis**

|  | ***RNF213* p.Arg4810Lys (N=10)** | **Wild type**  **(N=87)** | ***p value*** |  | ***RNF213* p.Arg4810Lys**  **(N=10)** | **Wild type**  **(N=87)** | ***p value*** |
| --- | --- | --- | --- | --- | --- | --- | --- |
| Age (years) | 68 (60–78) | 76 (69–84) | 0.126 | Previous tumour | 3 (30%) | 18 (23%) | 0.695 |
| Sex (female) | 2 (20%) | 28 (32%) | 0.719 | Living alone | 0 (0%) | 9 (19%) | 0.206 |
| Hypertension | 9 (90%) | 72 (82%) | 1.000 | Family history | 6 (60%) | 29 (41%) | 0.190 |
| Diabetes mellitus | 3 (30%) | 30 (34%) | 1.000 | Smoking  (current or former) | 6 (60%) | 49 (57%) | 1.000 |
| Dyslipidaemia | 4 (40%) | 43 (50%) | 0.741 | Heavy drinkers | 0 (0%) | 16 (18%) | 0.206 |
| Hyperuricemia | 1 (10%) | 7 (8.0%) | 0.987 | Previous CVD | 3 (30%) | 14 (15%) | 0.207 |
| Atrial fibrillation | 0 (0%) | 8 (9.2%) | 1.000 | Previous CI | 3 (30%) | 17 (17%) | 0.385 |
| Renal dysfunction | 1 (10%) | 8 (9.2%) | 1.000 | Previous ICH | 1 (10%) | 7 (8.0%) | 0.592 |
| Thyroid disease | 1 (10%) | 3 (3.4%) | 0.358 | Antiplatelet | 5 (50%) | 30 (32%) | 0.309 |
| PAD | 0 (0%) | 3 (3.4%) | 1.000 | Anticoagulant | 2 (20%) | 7 (18%) | 1.000 |

Statistically significant differences in background factors between the two groups were evaluated using Fisher’s exact test and the Mann–Whitney U test (for age). Median age [Quartile 1 and Quartile 3]. CAD, coronary artery disease; CI, cerebral infarction; ICH, intracranial haemorrhage. Max-IMT, maximum intima-media thickness; PAD, peripheral artery disease; CVD, cardiovascular disease

**Supplementary Table 4 Characteristics of intracranial and extracranial stenosis**

|  | **Kyorin cohort** | | **Validation cohort** | |
| --- | --- | --- | --- | --- |
| Stenosis cases (≥50%) | 209 | | 229 | |
| *RNF213* p.Arg4810Lys | Variant | Wild-type | Variant | Wild-type |
| N | 13 | 196 | 22 | 207 |
| **Stenosis/occlusion side** | | | | |
| ICAS-only | 5 (38%) | 123 (63%) | 10 (45%) | 149 (72%) |
| ECAS-only | 2 (15%) | 26 (13%) | 2 (9%) | 22 (11%) |
| ICAS + ECAS | 6 (46%) | 47 (24%) | 10 (45%) | 36 (17%) |
| **Anterior or posterior circulation** | | | | |
| Anterior circulation | 13 (100%) | 146 (74%) | 21 (95%) | 189 (91%) |
| -MCA | 5 (38%) | 56 (29%) | 14 (64%) | 83 (40%) |
| Posterior circulation | 5 (38%) | 85 (43%) | 5 (23%) | 86 (41%) |
| **Counts of stenosis/occlusion** | | | | |
| Single stenosis | 6 (38%) | 90 (46%) | 7 (32%) | 117 (57%) |
| Multiple stenosis | 7 (62%) | 102 (52%) | 15 (68%) | 90 (43%) |
| No. of tandem | | | | |
| 2 | 1 (7.7%) | 49 (25%) | 5 (23%) | 36 (17%) |
| ≥3 | 5 (38%) | 13 (6.6%) | 7 (32%) | 9 (4.3%) |

ICAS, intracranial artery stenosis; ECAS, extracranial artery stenosis; MCA, middle cerebral artery

**Supplementary Table 5 Association of ICAS / ECAS with *RNF213* p.Arg4810Lys in the validation cohort**

|  | **Frequency of *RNF213* p.Arg4810Lys** | | ***p* value** | **OR (95% CI)** |
| --- | --- | --- | --- | --- |
|  | **Case** | **Control** |  |  |
| ICAS-only | 10/159 (6.2％) | 1/131 (0.8%) | 0.007* | 10.08 (1.28–79.13) |
| ECAS-only | 2/24 (8.3%) | 1/131 (0.8%) | 0.026* | 10.56 (1.33–83.59) |
| ICAS + ECAS | 10/46 (22%) | 1/131 (0.8%) | 0.001* | 32.75 (4.02–267.12) |

Values are expressed as n (%). Statistically significant differences were determined using univariate logistic regression. *Statistical significance: *p*<0.05.

LAA, large artery atherosclerosis; ICAS, intracranial artery stenosis; ECAS, extracranial artery stenosis; CI, confidence interval; OR, odds ratio

**Supplementary Table 6 Single and multiple regression analyses of the risk factors for max-IMT in the validation cohort**

| **Risk factor** | **Single regression analysis** | | | **Multiple regression analysis** | | |
| --- | --- | --- | --- | --- | --- | --- |
|  | **β** | **SE** | ***p* value** | **β** | **SE** | ***p* value** |
| Age | 0.200 | 0.007 | 0.001* | 0.282 | 0.007 | <.0001* |
| Sex (female) | 0.179 | 0.169 | 0.003* | 0.181 | 0.165 | 0.002* |
| *RNF213* p.Arg4810Lys | 0.166 | 0.311 | 0.006* | 0.178 | 0.316 | 0.003* |
| Hypertension | 0.047 | 0.189 | 0.783 |  |  |  |
| Diabetes mellitus | 0.087 | 0.180 | 0.150 |  |  |  |
| Dyslipidaemia | 0.083 | 0.167 | 0.168 |  |  |  |
| Hyperuricaemia | 0.093 | 0.218 | 0.125 |  |  |  |
| Renal dysfunction | 0.034 | 0.309 | 0.569 |  |  |  |
| Peripheral artery disease | 0.091 | 0.497 | 0.131 |  |  |  |
| Thyroid disease | -0.112 | 0.250 | 0.064 |  |  |  |
| Previous CVD | 0.202 | 0.263 | 0.001* | 0.200 | 0.249 | 0.001* |
| Previous CI | 0.086 | 0.183 | 0.154 |  |  |  |
| Previous ICH | -0.062 | 0.364 | 0.332 |  |  |  |
| Previous tumour | -0.003 | 0.275 | 0.958 |  |  |  |
| Family history | -0.060 | 0.196 | 0.352 |  |  |  |
| Smoking (current or former) | -0.055 | 0.192 | 0.365 |  |  |  |
| Heavy drinkers | 0.100 | 0.262 | 0.128 |  |  |  |
| BMI | 0.038 | 0.019 | 0.556 |  |  |  |

Values are expressed as n (%). Statistically significant differences were determined using multivariate logistic regression.

SE, standard error; BMI, body mass index, CI, cerebral infarction; ICH, intracranial haemorrhage; CVD, cardiovascular disease

**Supplementary Figure 1 Flowchart of the validation cohort for the association of *RNF213* p.Arg4810Lys with ICAS / ECAS and max-IMT**

131 Control participants

391 Participants assessed for eligibility in the validation study

Excluded:

20 Cardioembolic stroke

11 Moyamoya disease

Excluded:

20 Cardioembolic stroke

11 Moyamoya disease

86 No ultrasonography

131 Without ICAS or ECAS

***RNF213* p.Arg4810Lys and max-IMT**

**274 Available cases**

***RNF213* p.Arg4810Lys and ICAS / ECAS**

**229 Available cases**

ICAS, intracranial artery stenosis; ECAS, extracranial artery stenosis; Max-IMT, maximum intima-media thickness

**Supplementary Figure 2 Max-IMT of the carotid artery by *RNF213* p.Arg4810Lys genotypes in the validation cohort**


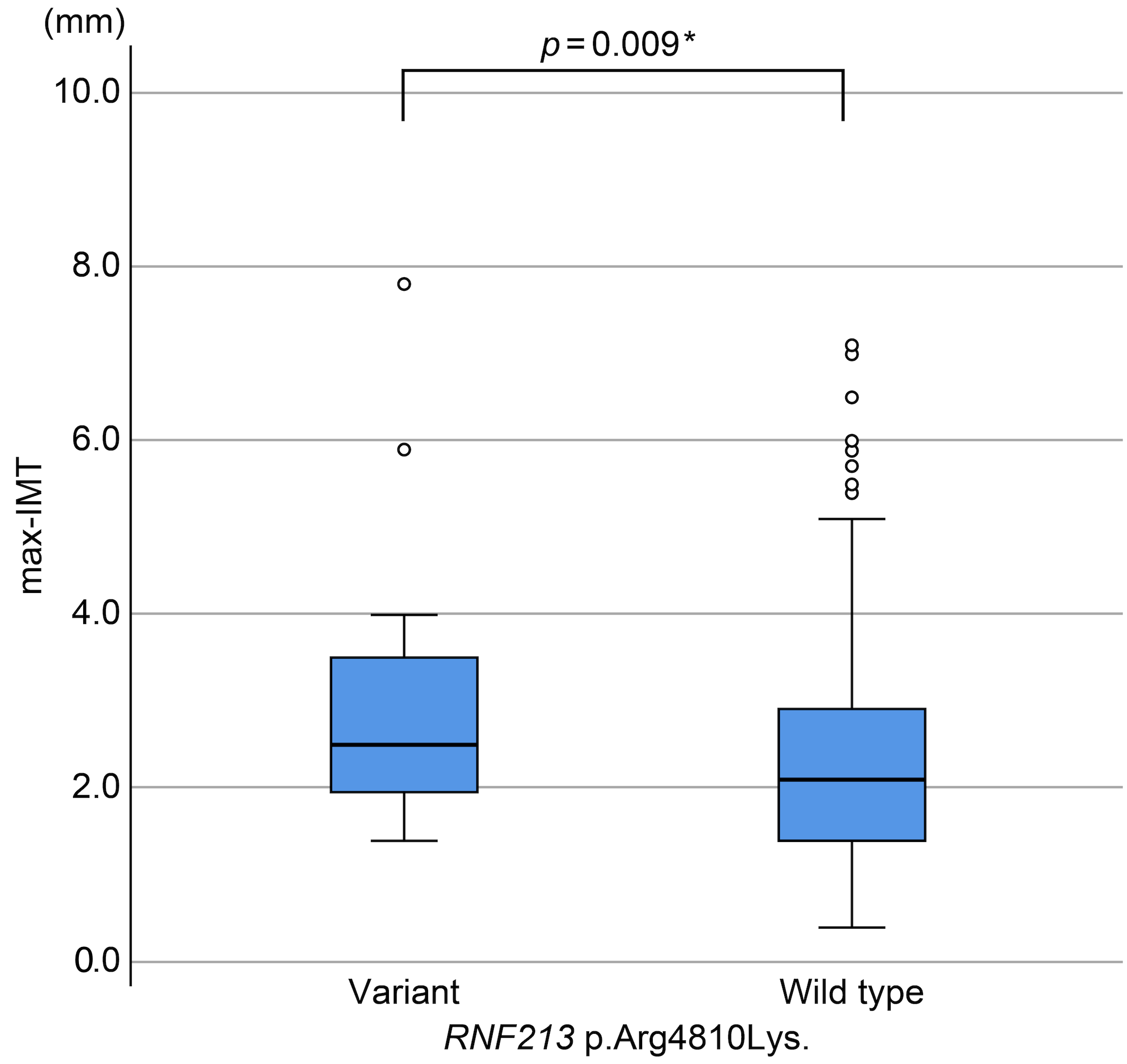
(mm)

**
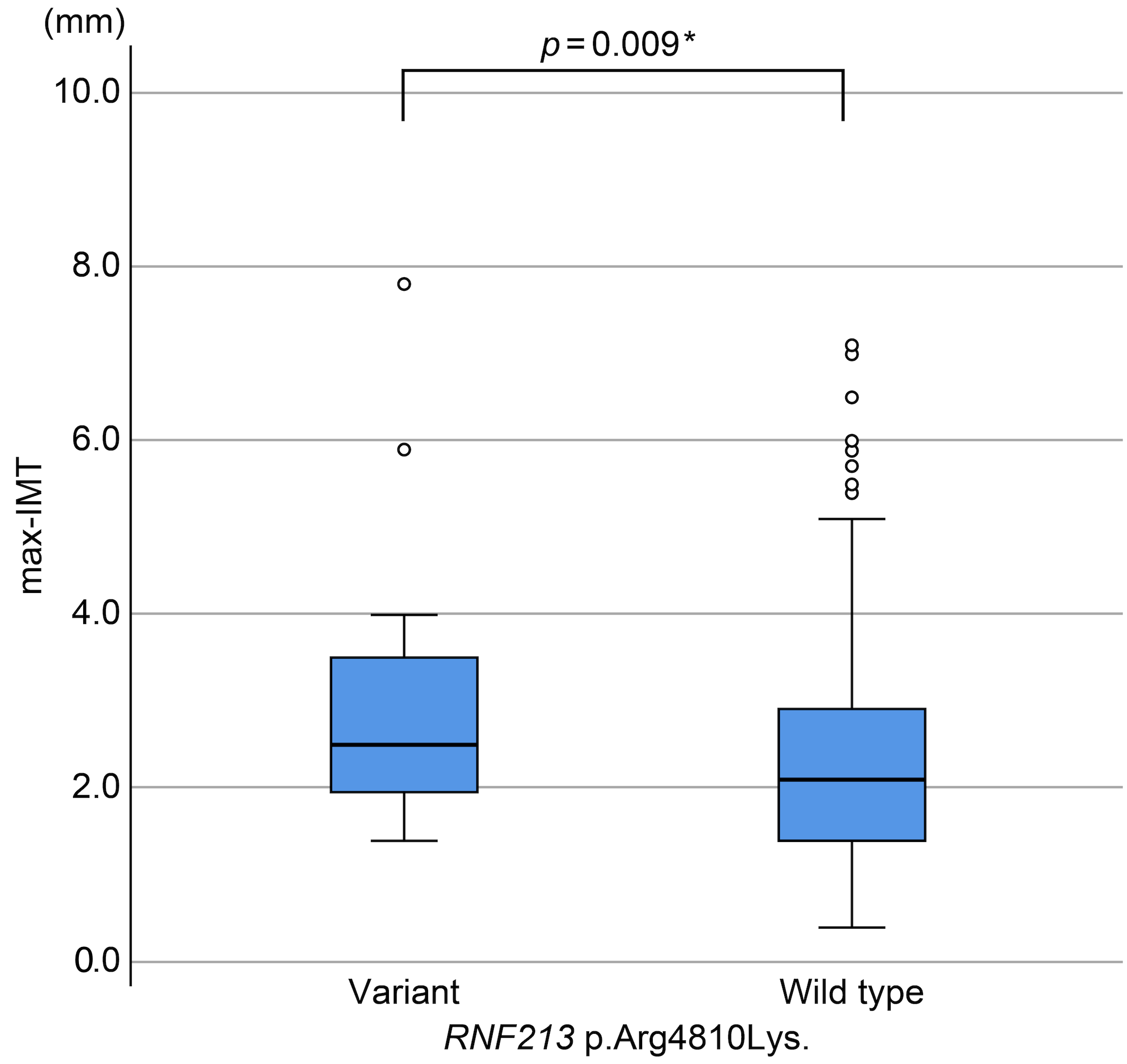
**

**Max-IMT of the carotid artery by *RNF213* p.Arg4810Lys genotypes in the validation cohort.** Box plot showing the significantly increased max-IMT of the carotid artery in the *RNF213* p.Arg4810Lys variant group (N=21) compared to that in the wild-type group (N=253). Statistical analysis was performed using the Mann–Whitney U test, with *p<0.05 considered significant.

Max-IMT, maximum intima-media thickness
